# Supplementary figures and images for: Pulmonary function analysis in cotton rats after respiratory syncytial virus infection
Source: PLoS One. 2020 Aug 10;15(8):e0237404. doi: 10.1371/journal.pone.0237404 (PMC7416943; doi:10.1371/journal.pone.0237404)

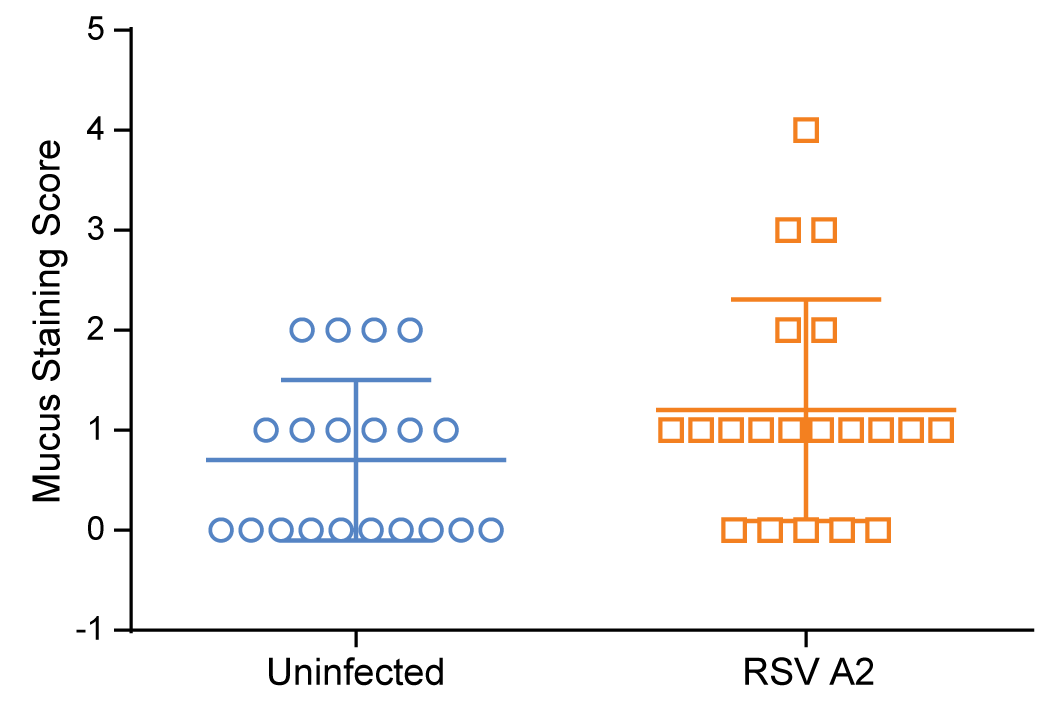

Supplement: S1 Fig — Quantification of PAS and Alcian blue positive (mucus) staining in bronchioles was performed using a semi-quantitative mucus scoring system. The mean and standard deviations are represented (n = 4/ group). There was no significant difference between groups (unpaired student T test, p value > 0.05). (TIF) [file pone.0237404.s001.tif]

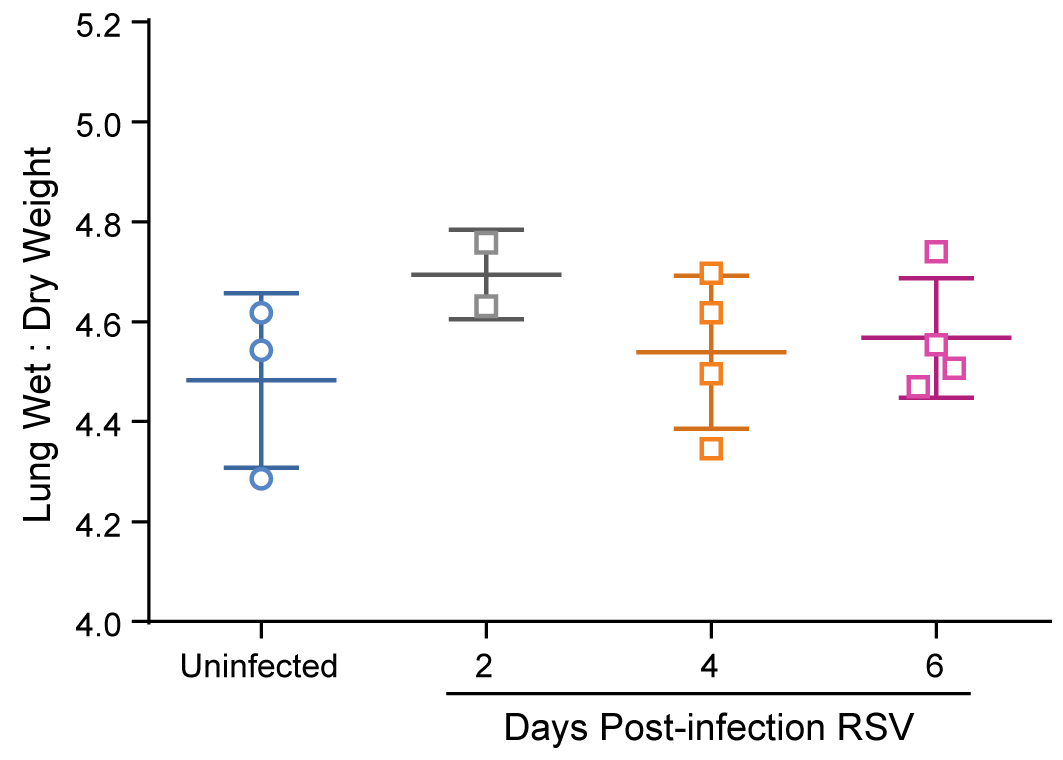

Supplement: S2 Fig — The lung wet: dry weigh ratios in uninfected cotton rats and cotton rats 2, 4 or 6 days post-RSV infection. Mean wet: dry weight ratios and standard deviations are represented. There is no significant difference when all groups were compared to one another by One-Way ANOVA, p > 0.05; uninfected (n = 3), 2DPI RSV (n = 2), 4DPI RSV (n = 4), 6 DPI RSV (n = 4). (TIF) [file pone.0237404.s002.tif]

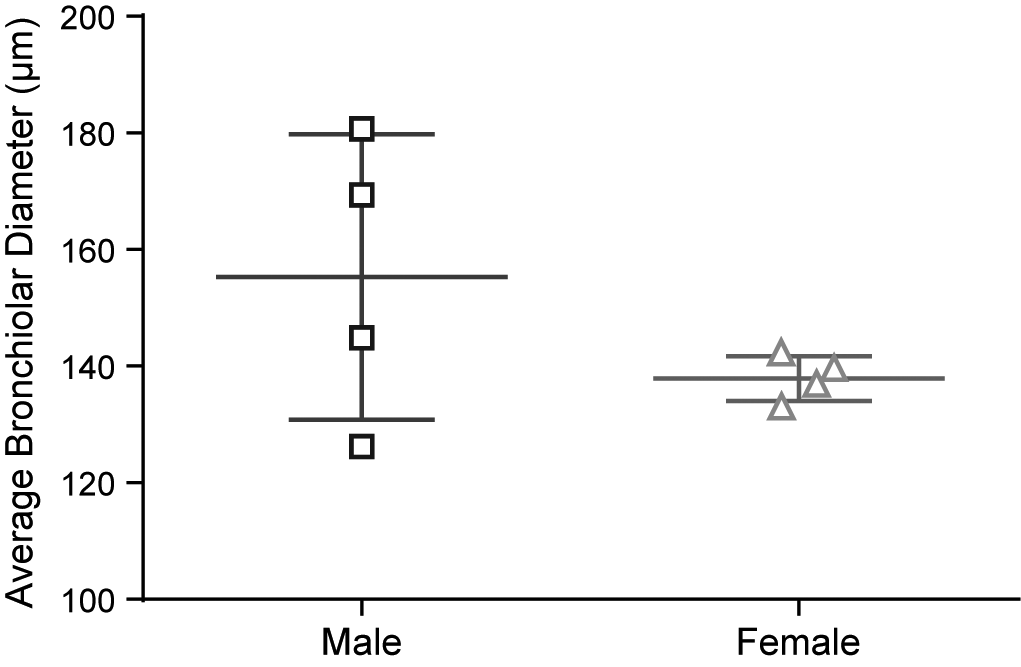

Supplement: S3 Fig — The diameter of an average of 43 small airways (bronchioles) per uninfected animal were measured microscopically. The mean and standard deviation for each group are represented. One-way ANOVA, p>0.05 (n = 4). (TIF) [file pone.0237404.s003.tif]

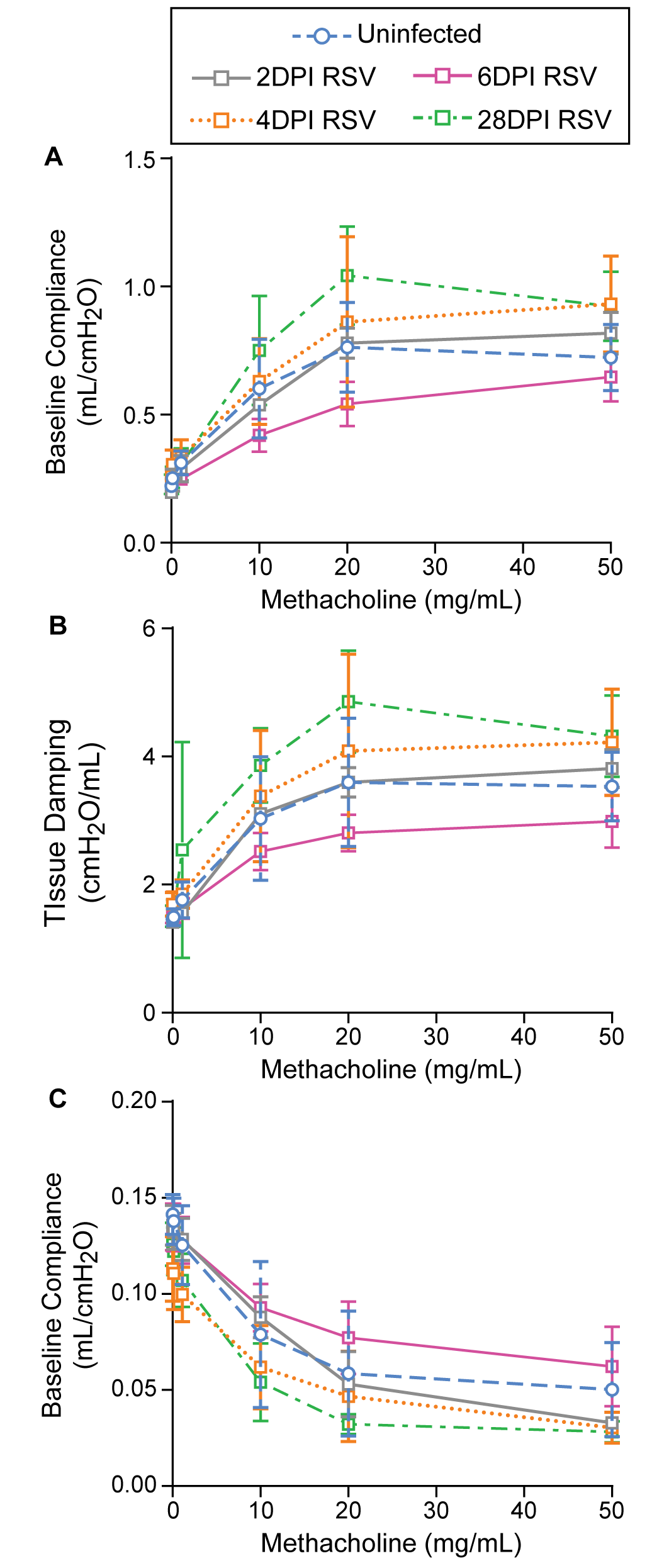

Supplement: S4 Fig — The mean and standard deviation for each group are represented. Figure a represents total airway resistance (R). Figure b represents tissue damping (G). Figure c represents dynamic pulmonary compliance (C). Uninfected (n ranges 7–6), 2DPI RSV (n = 3), 4DPI RSV (n ranges 7–6), 6DPI RSV (n = 5), 28DPI RSV (n ranges 6–5). (TIF) [file pone.0237404.s004.tif]

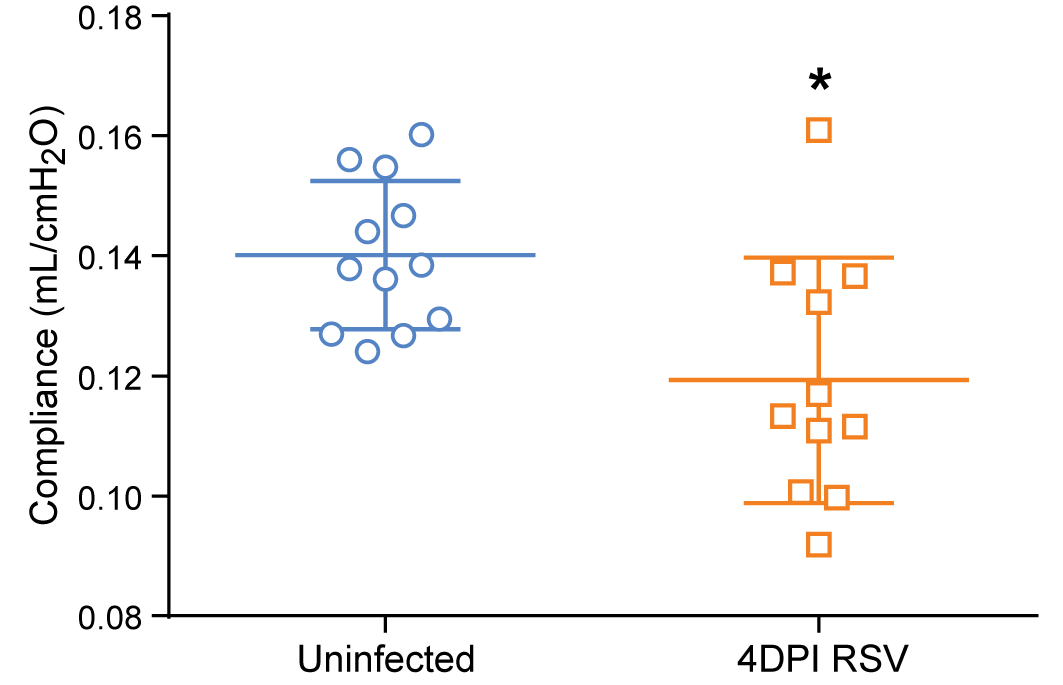

Supplement: S5 Fig — The mean and standard deviation for each group are represented. Both female and male cotton rats were presented in each group. Baseline pulmonary compliance was measured using forced oscillation technique. Asterisks indicate p< 0.05 by an unpaired two-tailed Student T-test. (TIF) [file pone.0237404.s005.tif]

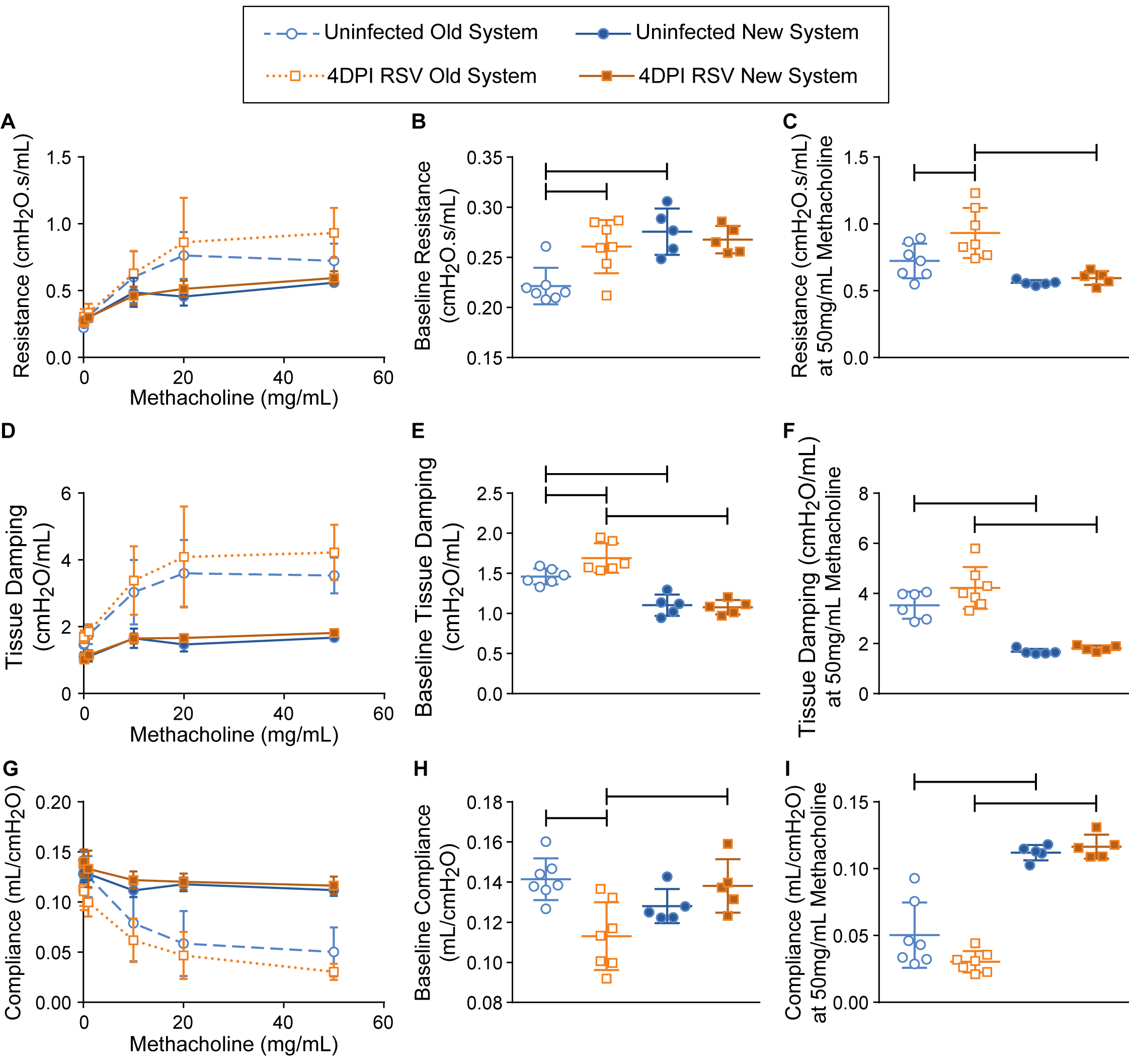

Supplement: S6 Fig — Pulmonary function was measured in female cotton rats using the FV-M2 (old) and FX3 (new) flexivent system in uninfected and 4 days post-infection (DPI) with RSV in female cotton rats. The mean and standard deviation for each group are represented. Figures a-c represent total airway resistance (R). Figures d-f represent tissue damping (G). Figures g-i represent dynamic pulmonary compliance (C). Figures a, d, and g represent the lung mechanic curves for R, G, and C. Figures b, e, and h represent the baseline measurements for R, G, and C. Figures c, f, and i represent the measurements obtained at 50mg/mL methacholine dosage for R, G, and C. Bars indicate p< 0.05 by One-way ANOVA; uninfected old system (n ranges 7–6), 4DPI RSV old system RSV (n ranges7-6), uninfected new system (n = 5), 4DPI RSV new system (n = 5). (TIF) [file pone.0237404.s006.tif]

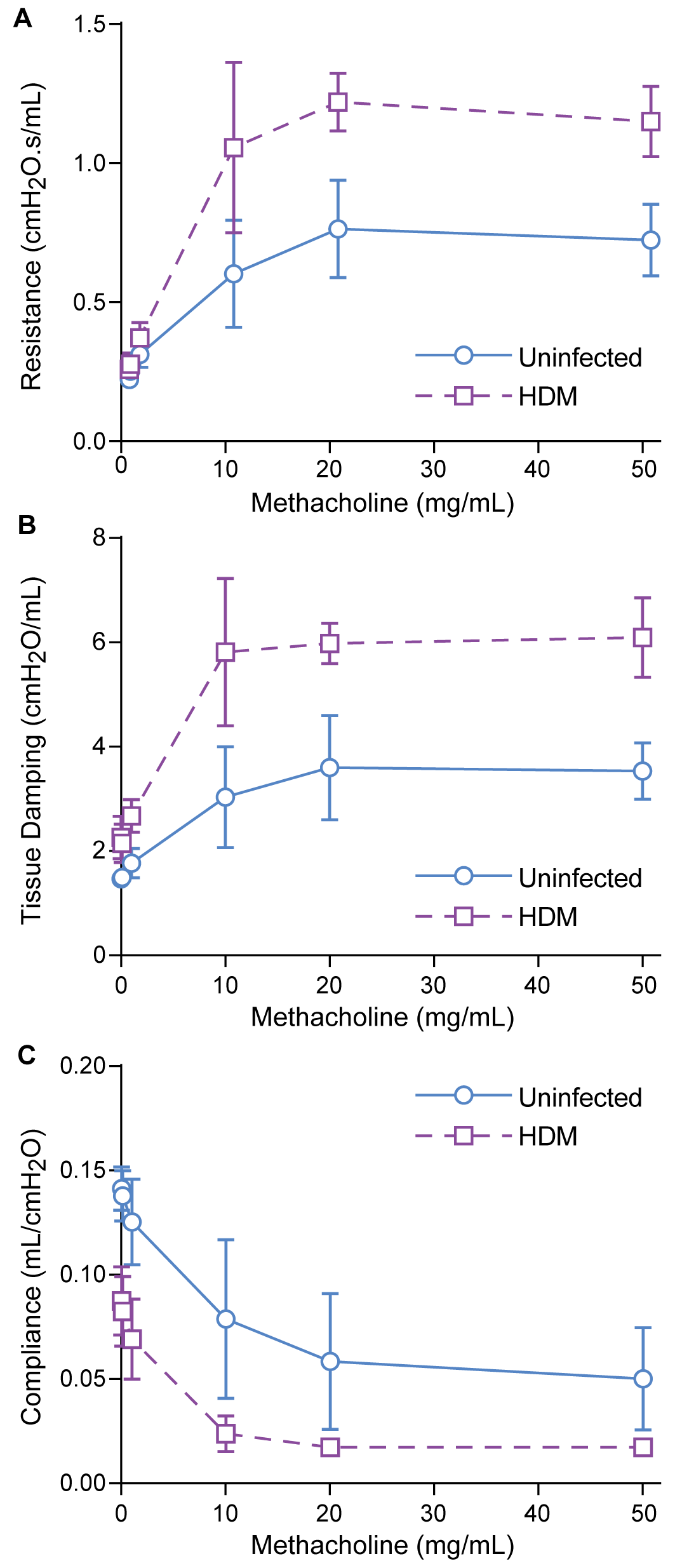

Supplement: S7 Fig — The mean and standard deviation for each group are represented. A) Measurement of total airway resistance (R). B) Measurement of issue damping (G). C) Measurement of dynamic pulmonary compliance (C). Uninfected (n ranges 7–6), HDM (n = 4). (TIF) [file pone.0237404.s007.tif]
