# Supplementary material for: Pulmonary function analysis in cotton rats after respiratory syncytial virus infection
Source: PLoS One. 2020 Aug 10;15(8):e0237404. doi: 10.1371/journal.pone.0237404 (PMC7416943; doi:10.1371/journal.pone.0237404)
Supplement: S1 Table — (DOCX) [file pone.0237404.s008.docx]

**S1 Table. Semi-Quantitative Histologic Inflammatory Scoring System.**

| **Characteristic** | **Description** | **Grade** |
| --- | --- | --- |
| **Quantity of Peribronchiolar infiltrates** | 0% of bronchioles with peribronchiolar infiltrates | 0 |
|  | 0-25% of bronchioles with peribronchiolar infiltrates | 1 |
|  | 25-50% of bronchioles with peribronchiolar infiltrates | 2 |
|  | 50-75% of bronchioles with peribronchiolar infiltrates | 3 |
|  | >75% of bronchioles with peribronchiolar infiltrates | 4 |
| **Severity of Peribronchiolar infiltrates** | No cells | 0 |
|  | interuppted cuff | 1 |
|  | Complete cuff, <5 cell layers thick | 2 |
|  | Complete cuff, >5 cell layers thick | 3 |
| **Quantity of Bronchiolar infiltrates** | 0% of bronchioles with bronchiolar epithelium infiltrates | 0 |
|  | 0-25% of bronchioles with bronchiolar epithelium infiltrates | 1 |
|  | 25-50% of bronchioles with bronchiolar epithelium infiltrates | 2 |
|  | 50-75% of bronchioles with bronchiolar epithelium infiltrates | 3 |
|  | >75% of bronchioles with bronchiolar epithelium infiltrates | 4 |
| **Severity of Bronchiolar infiltrates** | No infiltrating cells within the bronchiolar epithelium | 0 |
|  | 0-10% of the bronchiolar epithelium has inflammatory infiltrates | 1 |
|  | 10-33% of the bronchiolar epithelium has inflammatory infiltrates | 2 |
|  | 33-66% of the bronchiolar epithelium has inflammatory infiltrates | 3 |
|  | >66% of the bronchiolar epithelium has inflammatory infiltrates | 4 |
| **Quantity of Perivascular infiltrates** | 0% of bronchioles with peribronchiolar infiltrates | 0 |
|  | 0-25% of bronchioles with peribronchiolar infiltrates | 1 |
|  | 25-50% of bronchioles with peribronchiolar infiltrates | 2 |
|  | 50-75% of bronchioles with peribronchiolar infiltrates | 3 |
|  | >75% of bronchioles with peribronchiolar infiltrates | 4 |
| **Severity of Perivascular infiltrates** | No cells | 0 |
|  | interuppted cuff | 1 |
|  | Complete cuff, <5 cell layers thick | 2 |
|  | Complete cuff, >5 cell layers thick | 3 |
| **Quantity of Interstitial infiltrates** | 0% interstitial infiltrates | 0 |
|  | 0-10% interstitial infiltrates | 1 |
|  | 10-33% interstitial infiltrates | 2 |
|  | 33-66% interstitial infiltrates | 3 |
|  | >66%interstitial infiltrates | 4 |
| **Quantity of Alveolar infiltrates** | 0% of alveolar spaces with inflammatory infiltrates | 0 |
|  | 0-10% of alveolar spaces with inflammatory infiltrates | 1 |
|  | 10-33% of alveolar spaces with inflammatory infiltrates | 2 |
|  | 33-66% of alveolar spaces with inflammatory infiltrates | 3 |
|  | >66% of alveolar spaces with inflammatory infiltrates | 4 |
