# Supplementary material for: Pulmonary function analysis in cotton rats after respiratory syncytial virus infection
Source: PLoS One. 2020 Aug 10;15(8):e0237404. doi: 10.1371/journal.pone.0237404 (PMC7416943; doi:10.1371/journal.pone.0237404)
Supplement: S2 Table — (DOCX) [file pone.0237404.s009.docx]

**S2 Table. Semi-Quantitative Histologic Mucus Scoring System.**

| Characteristic | Description | Grade |
| --- | --- | --- |
| PAS Staining | no PAS positive cells | 0 |
|  | <5% PAS positive cells | 1 |
|  | 5-10% PAS positive cells | 2 |
|  | 10-25% positive cells | 3 |
|  | >25% PAS positive cells | 4 |
