# Supplementary material for: Pulmonary function analysis in cotton rats after respiratory syncytial virus infection
Source: PLoS One. 2020 Aug 10;15(8):e0237404. doi: 10.1371/journal.pone.0237404 (PMC7416943; doi:10.1371/journal.pone.0237404)
Supplement: S3 Table — (DOCX) [file pone.0237404.s010.docx]

**S3 Table. Collar sensor clip pulse oximetry measurements.**

|  | Uninfected | 2DPI RSV | 4DPI RSV | 6DPI RSV |
| --- | --- | --- | --- | --- |
| Peripheral blood oxygenation (S_p_O_2_) | 99.05 (0.40) | 97.13 (3.86) | 99.21 (0.10) | 99.18 (0.21) |
| Heart Rate (beats/ minute) | 525.30 (18.47) | 511.60 (58.32) | 557.90 (18.65) | 507.50 (32.60) |
| Breath Rate (breaths/ minute) | 96.86 (10.67) | 118.80 (44.72) | 101.00 (13.68) | 79.42 (9.60) |
| Pulse Distention (µm) | 508.10 (129.90) | 445.80 (26.96) | 426.50 (103.00) | 391.60 (129.10) |

The mean and standard deviation for each group are represented. N=4.
