# Supplementary material for: Pulmonary function analysis in cotton rats after respiratory syncytial virus infection
Source: PLoS One. 2020 Aug 10;15(8):e0237404. doi: 10.1371/journal.pone.0237404 (PMC7416943; doi:10.1371/journal.pone.0237404)
Supplement: S4 Table — (DOCX) [file pone.0237404.s011.docx]

**S4 Table.** **Thigh sensor clip pulse oximetry measurements.**

|  | Uninfected | 2DPI RSV | 4DPI RSV | 6DPI RSV |
| --- | --- | --- | --- | --- |
| Peripheral blood oxygenation (S_p_O_2_) | 98.33 (0.64) | 98.95 (0.27) | 98.37 (0.84) | 97.87 (1.08) |
| Heart rate (beats/ minute) | 501.60 (13.08) | 487.6 (63.65) | 532.0 (33.75) | 495.9 (30.09) |
| Breath rate (breaths/ minute) | 99.54 (16.94) | 96.06 (20.97) | 104.3 (8.96) | 98.55 (39.41) |
| Pulse distention (µm) | 43.43 (17.99) | 67.19 (68.21) | 50.04 (32.22) | 49.46 (11.73) |
| Breath distention (µm) | 43.52 (42.27) | 23.34 (17.51) | 19.91 (5.054) | 24.80 (7.54) |

The mean and standard deviation for each group are represented. N=4.
