# Supplementary material for: Pulmonary function analysis in cotton rats after respiratory syncytial virus infection
Source: PLoS One. 2020 Aug 10;15(8):e0237404. doi: 10.1371/journal.pone.0237404 (PMC7416943; doi:10.1371/journal.pone.0237404)
Supplement: S5 Table — (DOCX) [file pone.0237404.s012.docx]

**S5 Table. Arterial blood gas measurements.**

|  | Uninfected | 2DPI RSV | 4DPI RSV |
| --- | --- | --- | --- |
| P_a_O_2_ | 142.70 (39.12) | 114.80 (15.24) | 116.20 (5.0) |
| P_a_CO_2_ | 25.97 (9.03) | 32.18 (4.92) | 31.40 (2.31) |
| pH | 7.29 (0.1678) | 7.27 (0.074) | 7.26 (0.07) |
| P/F ratio | 679.40 (186.3) | 546.40 (72.57) | 553.20 (23.79) |

The mean and standard deviation for each group are represented. Uninfected n=3, 2DPI RSV n=4, 4DPI RSV n=6.
